# Quantification and correlates of tuberculosis stigma along the tuberculosis testing and treatment cascades in South Africa: a cross-sectional study

**DOI:** 10.1186/s40249-020-00762-8

**Published:** 2020-10-22

**Authors:** Dana Bresenham, Aaron M. Kipp, Andrew Medina-Marino

**Affiliations:** 1 Research Unit, Foundation for Professional Development, 10 Rochester Rd, Vincent, East London, South Africa; 2 Vanderbilt Institute for Global Health, Vanderbilt University, Nashville, TN USA; 3 Department of Medicine, Division of Epidemiology, Vanderbilt University Medical Center, Nashville, TN USA; 4 Desmond Tutu HIV Centre, Men’s Health Division, University of Cape Town, Cape Town, South Africa; 5 Department of Public Health, East Carolina University, 115 Heart Drive, Ste #2219, Greenville, NC 27858-4353 USA

**Keywords:** Tuberculosis, Stigma, Tuberculosis patient, Tuberculosis presumptive, Tuberculosis cascade, South africa

## Abstract

**Background:**

South Africa has one of the world’s worst tuberculosis (TB) (520 per 100 000 population) and TB-human immunodeficiency virus (HIV) epidemics (~ 56% TB/HIV co-infected). While individual- and system-level factors influencing progression along the TB cascade have been identified, the impact of stigma is underexplored and underappreciated. We conducted an exploratory study to 1) describe differences in perceived community-level TB stigma among community members, TB presumptives, and TB patients, and 2) identify factors associated with TB stigma levels among these groups.

**Methods:**

A cross sectional study was conducted in November 2017 at public health care facilities in Buffalo City Metro (BCM) and Zululand health districts, South Africa. Community members, TB presumptives, and TB patients were recruited. Data were collected on sociodemographic characteristics, TB knowledge, health and clinical history, social support, and both HIV and TB stigma. A validated scale assessing perceived community TB stigma was used. Univariate and multivariate linear regression models were used to describe differences in perceived community TB stigma by participant type and to identify factors associated with TB stigma.

**Results:**

We enrolled 397 participants. On a scale of zero to 24, the mean stigma score for TB presumptives (14.7 ± 4.4) was statistically higher than community members (13.6 ± 4.8) and TB patients (13.3 ± 5.1). Community members from Zululand (*β* = 5.73; 95% *CI* 2.19, 9.72) had higher TB stigma compared to those from BCM. Previously having TB (*β* = − 2.19; 95% *CI* − 4.37, 0.0064) was associated with reduced TB stigma among community members. Understanding the relationship between HIV and TB disease (*β* = 2.48; 95% *CI* 0.020, 4.94), and having low social support (*β* = − 0.077; 95% *CI* − 0.14, 0.010) were associated with increased TB stigma among TB presumptives. Among TB Patients, identifying as Black African (*β* = − 2.90; 95% *CI* − 4.74, − 1.04) and knowing the correct causes of TB (*β* = − 2.93; 95% *CI* − 4.92, − 0.94) were associated with decreased TB stigma, while understanding the relationship between HIV and TB disease (*β* = 2.48; 95% *CI* 1.05, 3.90) and higher HIV stigma (*β* = 0.32; 95% *CI* 0.21, 0.42) were associated with increased TB stigma.

**Conclusions:**

TB stigma interventions should be developed for TB presumptives, as stigma may increase initial-loss-to-follow up. Given that stigma may be driven by numerous factors throughout the TB cascade, adaptive stigma reduction interventions may be required.

## Background

In 2018, an estimated 10 million individuals developed tuberculosis (TB) disease globally, of which an estimated 1.5 million died; human immunodeficiency virus (HIV) was the leading cause of death [1]. South Africa has the world’s highest TB incidence (520 per 100 000 population) and HIV-TB co-epidemic (~ 56% HIV co-infected) [1]. Historically, the South African National TB Programme (SA-NTP) focused on treatment success rates to the detriment of upstream indicators, such as case finding, linkage-to-care and treatment initiation, which are not reflected in the SA-NTP treatment success indicator. This was powerfully evident from a recent analysis of the South African TB Cascade which estimated 30% of cases were missed, not diagnosed, or never initiated treatment [2]. When including the 17% of cases not successfully completing treatment, ~ 50% of those with TB in South Africa are not successfully identified, cared for, or treated. While individual- and system-level barriers and facilitators impacting progression along the TB cascade have been identified [3–6], the impact of stigma on the TB care cascade, from seeking care and testing to diagnosis, from treatment initiation to successful completion, is underexplored and underappreciated.

Acknowledging health related stigma (e.g., TB-related stigma) as a social process highlights the interaction between infected and uninfected individuals. The three predominant ways stigma is perpetrated by individuals towards others with a stigmatizing condition include: (1) discrimination, (2) prejudice, and (3) stereotyping [7, 8]. Among stigmatized individuals, there are three major domains of stigma: anticipated stigma (anticipated/perceived prejudice or discrimination in the community towards those with TB), enacted stigma (experiencing discrimination due to TB status), and internalized stigma (feeling shame or blame due to TB status) [9, 10]. In contrast, perceived stigma refers to the perceived level of stigma in the community (prejudice, discrimination) by both individual community members and patients. When stigma is propagated and experienced throughout communities, it may directly impact the health behaviors and outcomes of those with the stigmatizing condition. More importantly it may impact the development and successful implementation of interventions geared towards reducing the disease burden of the stigmatized condition. This is especially the case of TB [11–13]. Historically, TB has been stigmatized due to a lack of knowledge surrounding its direct cause, fear of infection, or association with marginalized groups (i.e., the poor, immigrants, or hygienically “dirty people”) [14–17]. Moreover, due to the biological, epidemiological and social interactions between TB and HIV/AIDS, TB increasingly became more stigmatized, especially among populations with high rates of TB and HIV co-infections [16–20].

Qualitative studies from South Africa and elsewhere continue to identify stigma as a barrier to progression along the TB care cascade [19, 21–23]. Less frequently, quantitative studies have explored the impact of TB stigma on the TB care cascade [24, 25]. Studies focusing on the impact of TB stigma and delayed health seeking behavior have presented contradicting results, were all clinic-based, cross-sectional studies, and excluded “missed” TB cases who were never tested [26–29]. Additionally, studies of TB stigma on treatment adherence and completion have also been replete with contradictory results despite using prospective designs [29–38]. Lack of validated scales that distinguish between and measure the different domains of stigma could provide one plausible explanation for these contradictory results. The few studies that have included the evaluation of the different domains of TB stigma suggest that they may differentially impact various phases of the care cascade [29, 32]. Furthermore, no studies have explored how the impact of stigma may change with progression through the care cascade*.*

The importance of understanding and addressing individual-level TB stigma is evident as it hinders retention and progression along the cascade [12, 13, 16, 39–42]. Furthermore, given that stigma originates and emanates from multiple levels outside the individual (i.e., family, community, institutions), it is also essential to understand TB-related stigma at a community level [32]. Consequently, additional quantitative work is needed to assist stakeholders, such as TB programme implementers or policy makers, to understand the magnitude of the problem and stigma’s impact in specific settings or distinct subpopulations. Towards this, this analysis aimed to 1) describe the differences in perceived community level TB stigma among community members, TB presumptives, and TB patients, and 2) to identify factors associated with TB stigma levels among these groups.

## Methods

### Study design and setting

Data was utilized from a convenience sample used to validate two TB stigma scales. The cross sectional study was conducted in November 2017 utilizing interviewer-administered questionnaires via electronic tablets at public health care facilities in two districts in South Africa—Buffalo City Metro (BCM), Eastern Cape Province, and Zululand, KwaZulu Natal Province. Districts were selected based on their TB incidence, representation of the two ethnic groups with the highest TB burden (isiZulu and isiXhosa), and convenience access due to being President’s Emergency Plan for AIDS Relief (PEPFAR) supported districts. In 2016, BCM had a TB incidence of 743 per 100 000 population, and Zululand had a TB incidence of 683 per 100 000 [43]. The predominant language spoken in BCM is Xhosa, while the predominant language spoken in Zululand is Zulu; English and Afrikaans are also spoken in both districts. In both BCM and Zululand, TB is the leading cause of death among people aged 25–64 years [43].

### Sampling and study population

Three types of participants were recruited from 11 clinics (six clinics in BCM and five clinics in Zululand)—community members, TB presumptives, and TB patients. Clinics with the highest TB case load per district were selected for the study. Community members were defined as anyone attending a health facility for services other than TB. TB presumptives were defined as patients attending the clinic who screened positive for TB symptoms and submitted a sputum sample to be tested for TB. TB patients were defined as patients attending the clinic who have been on TB treatment for more than one month. Eligibility criteria included: (1) fluency in IsiZulu, IsiXhosa, English or Afrikaans, (2) resident in the catchment area of a study clinic, and (3) age ≥ 18 years. A convenience sample of approximately 450 participants was purposefully selected in a (1) 1:1:1 ratio of community members, TB presumptives, and TB patients, (2) equal representation of men and women, and (3) equal representation of the four languages to ensure a diverse representation for validating and assessing TB stigma.

### Data collection and measures

All questionnaires were developed in English, translated into local languages, back translated to ensure translation accuracy, and piloted in specific communities to ensure meaning was retained. Questionnaires included items on sociodemographic characteristics, TB knowledge, health and clinical history, social support, and both HIV and TB stigma (Additional file 1).

*Sociodemographic items* included community type, language, age, gender, race, level of education, marital status, employment status, and income. *TB Knowledge items* included open-ended questions about causes of TB and TB/HIV interaction. Responses regarding knowledge of what causes TB were coded on a three-point scale. *Health and clinical history* included knowing TB contacts, ever having TB, HIV status, and depression (measured using the PHQ-9 but excluding the final question on suicidal ideation) [44]. *Social support* was measured using the multidimensional scale of perceived social support [45]. Responses to individual items were summed to create a social support score, such that higher scores indicate more social support. *Perceived HIV stigma* was measured among TB patients using the 12-item attributable HIV stigma scale developed and validated in South Africa [46]. Given HIV co-infection in > 50% of TB patients in the study sites, this was more appropriate than asking TB patients directly how they feel about people with HIV. In contrast, *community HIV stigma* was measured among TB presumptives and community members using the 9-item Kalichman HIV stigma scale also developed and validated in South Africa [47]. For each scale, individual responses were summed to create a stigma score, with higher summed scores indicating higher levels of HIV stigma.

*TB stigma* was the dependent variable of interest, measured using the Van Rie perceived community TB stigma scale with a four-point Likert-type response ranging from strongly disagree (coded as 0) to strongly agree (coded as 3). For each scale, individual responses were summed to create a stigma score, with higher summed scores indicating higher levels of TB stigma [48]. The original scale contained 11 items, of which three were dropped following validation in the South African context due to poor performance on cognitive testing and/or factor analysis [49]. The remaining eight items had a Cronbach alpha ranging from 0.86 to 0.93 across participant types, indicating good reliability.

### Survey quality

Surveys were developed and piloted in study communities with cognitive interviews among community member, TB presumptives, and TB patients. Minor changes were made to optimize the understanding of items within our target study population. Validated questionnaires were used to measure stigma to enhance result validity. Data from surveys were collected and captured onto REDCap, an Advanced Real-Time Electronic Data System (EDS). The EDS provides an integrated, real-time data platform to monitor data entries and provide real-time data quality checks in order to reduce data queries.

### Data analysis

Data from a study aimed at validating TB stigma scales were used to perform an exploratory analysis to (1) examine differences in stigma between community members, TB presumptives, and TB patients, and (2) identify factors associated with TB stigma among these groups. A multivariable linear regression model was used to estimate differences in mean TB stigma scores between the three participant types while adjusting for gender, age, race, and enrollment district. Next, three parallel analyses were conducted to identify factors associated with higher TB stigma within each participant group. First, crude linear regression was used to estimate associations between TB stigma and each factor individually. Due to the exploratory nature of this analysis, factors whose *P*-value was ≤ 0.25 were included together in a multivariable model to adjust for potential confounding. District and gender were included regardless of the *P*-value. Because depression was assessed only among TB patients and presumptives, multivariable models were first run excluding depression (even if *P* ≤ 0.25) and then repeated to include depression if *P* ≤ 0.25. All model coefficients are interpreted as the mean difference in TB stigma scores between the index and referent categories for each factor of interest adjusting for other covariates in the model. Given the small sample sizes for each group and the potential for many covariates within a model, we report results where the *P*-value is < 0.15 to minimize Type II errors. Missing data was very rare, thus complete case analyses were conducted. For regression models, stigma scores were normally distributed and residual plots and tests for equality of variance demonstrated no major departure from homoscedasticity, indicating linear regression is appropriate. Descriptive and multivariable analyses were performed with STATA 13.1 software (StataCorp, College Station, Texas, USA).

### Ethics approval

Ethics approval was obtained from the Foundation for Professional Development Research Ethics Committee (Registration No. REC-03711-033-RA; Approval No. REC-2-2017). Permission was provided by the Eastern Cape and KwaZulu Natal Provincial research committees, and Departments of Health for BCM and Zululand Districts. Written informed consent was obtained from study participants prior to data collection.

## Results

### Participant characteristics

A total of 397 participants were enrolled; 163 community members, 127 TB presumptives, and 107 were patients (Table 1). Complete data were available for 392 of the 397 participants; one community member did not respond to community type; two community members and one TB presumptive did not respond to TB history, and one TB patient did not respond to employment status. The majority of participants were black African (71.5%), single (65.7%), unemployed (57.1%), residing in a small town (73.2%), with a primary level of education (57.9%), and making less than ZAR 5000 (~ USD 277) per month (75.6%). Overall, 65 (16.5%) participants reported ever having had TB and 138 (34.7%) self-reported having HIV. Characteristics that varied significantly between participant types included: language, income, HIV status, and social support (Table 1). Language and district were highly correlated, with Afrikaans, English, and Xhosa speaking participants predominantly enrolled in BCM while Zulu speaking participants were enrolled solely in Zululand.

**Table 1:** Sociodemographic and clinical characteristics of study participants, 397 (100%)

Characteristic	Total (397, 100%)	Community members (163, 41.0%)	TB presumptives (127, 32.0%)	TB patients (107, 27.0%)	*P*-value
Sociodemographic data					
District					
BCM	275 (69.3)	121 (74.2)	86 (67.7)	68 (63.6)	0.16
Zululand	122 (30.7)	42 (25.8)	41 (32.3)	39 (36.5)	
Language					
English	77 (19.4)	39 (23.9)	29 (22.8)	9 (8.4)	*0.01*
IsiXhosa	121 (30.5)	43 (26.4)	39 (30.7)	39 (36.5)	
Afrikaans	77 (19.4)	39 (23.9)	18 (14.2)	20 (18.7)	
IsiZulu	122 (30.7)	42 (25.8)	41 (32.3)	39 (36.5)	
Gender					
Male	188 (47.4)	78 (47.8)	57 (44.9)	53 (49.5)	0.77
Female	209 (52.6)	85 (52.2)	70 (55.1)	54 (50.5)	
Age, mean (IQR)	33 (17)	31 (16)	34 (17)	35 (16)	0.43
Race					
Black	284 (71.5)	106 (65.0)	97 (76.4)	81 (75.7)	0.056
Non-black	113 (28.5)	57 (35.0)	30 (23.6)	26 (24.3)	
Relationship status					
Union	136 (34.3)	62 (38.0)	38 (30.0)	36 (33.6)	0.35
Non-union	261 (65.7)	101 (62.0)	89 (70.0)	71 (66.4)	
Employment status					
Employed	170 (42.9)	74 (45.4)	49 (38.6)	47 (44.3)	0.48
Unemployed	226 (57.1)	89 (54.6)	78 (61.4)	59 (55.7)	
Community type					
Rural/farming area	106 (26.8)	37 (22.8)	34 (26.8)	35 (32.7)	0.20
Small town	290 (73.2)	125 (77.2)	93 (73.2)	72 (67.3)	
Education					
12th grade and above	167 (42.1)	74 (45.4)	54 (42.5)	39 (36.5)	0.34
Below 12th grade	230 (57.9)	89 (54.6)	73 (57.5)	68 (63.5)	
Income					
< ZAR 5000	300 (75.6)	112 (68.7)	102 (80.3)	86 (80.4)	*0.03*
≥ ZAR 5000	97 (24.4)	51 (31.3)	25 (19.7)	21 (19.6)	
TB knowledge					
Causes of TB**					
Poor understanding	171 (43.1)	63 (38.6)	66 (52.0)	42 (39.3)	0.07
Mixed understanding	66 (16.6)	26 (16.0)	16 (12.6)	24 (22.4)	
Good understanding	160 (40.3)	74 (45.4)	45 (35.4)	41 (38.3)	
TB/HIV knowledge					
HIV increase chances of TB					
Yes	340 (85.6)	142 (87.1)	108 (85.0)	90 (84.1)	0.77
No	57 (14.4)	21 (12.9)	19 (15.0)	17 (15.9)	
TB increase chances of HIV					
Yes	182 (45.8)	74 (45.4)	61 (48.0)	47 (43.9)	0.81
No	215 (54.2)	89 (54.6)	66 (52.0)	60 (56.1)	
Clinical data					
TB contacts					
Yes	153 (38.5)	54 (33.1)	59 (46.5)	40 (37.4)	0.07
No	244 (61.5)	109 (66.9)	68 (53.5)	67 (62.6)	
Ever had TB					
Yes	65 (16.5)	21 (13.0)	23 (18.3)	21 (19.6)	0.30
No	329 (83.5)	140 (87.0)	103 (81.7)	86 (80.4)	
HIV status*					
Positive	138 (34.7)	36 (22.1)	49 (38.6)	53 (49.5)	*0.00*
Negative	196 (49.4)	96 (58.9)	59 (46.4)	41 (38.3)	
Unknown/no test	63 (15.9)	31 (19.0)	19 (15.0)	13 (12.2)	
HIV stigma, mean ± SD					
Community and presumptive range: 0–27		4.48 ± 3.4	5.55 ± 4.1	–	
Patient range: 0–42		–	–	17.27 ± 7.3	
Mental health, mean ± SD					
Range: 0–24		–	5.43 ± 5.1	4.94 ± 4.6	
Social support, mean ± SD					
Range: 12–84		67.9 ± 9.3	63.9 ± 11.5	63.5 ± 11.0	*0.00*

*IQR* interquartile range, *SD* standard deviation, *TB* tuberculosis, *BCM* Buffalo City Metro

* HIV status was asked to the subset of the population who responded ‘yes’ to the self-reported HIV test

** Good understanding denotes participants who only identified accurate causes of TB (i.e. bacteria). Mixed understanding denotes participants who identified accurate and mis-informed causes of TB (i.e. infection and witchcraft). Poor understanding denotes participants who only identified incorrect causes of TB (i.e. exposure to the cold)

### Stigma scale responses

TB stigma scores were normally distributed. On a scale of 0–24, mean stigma scores were 13.6, 14.7, and 13.3 for community members, TB presumptives, and TB patients, respectively (Table 2). Regression analysis revealed the mean stigma score for TB presumptives was statistically higher than community members (score difference: 1.21, 95% *CI* 0.14, 2.27) and TB patients (score difference: 1.64, 95% *CI* 0.46, 2.83) (Table 2). There was no statistical difference between TB patients and community members (score difference: 0.44, 95% *CI* − 0.69, 1.57).

**Table 2:** Tuberculosis stigma scores by participant type

TB stigma	Community members	TB presumptives	TB patients	*P*-value
Perceived community TB Stigma (mean ± SD)				
Range: 0–24	13.6 ± 4.8	14.7 ± 4.4	13.3 ± 5.1	*0.043*

Adjusted comparisons*	Stigma score difference	Std. Err	95% *CI*	
Presumptive vs community member	1.21	0.54	(0.14, 2.27)	
Presumptive vs patients	1.64	0.60	(0.46, 2.83)	
Patient vs community member	0.44	0.58	(− 0.69, 1.57)	

^*^Adjusted for gender, age, race, and district

### Correlates of TB stigma among community members

Correlates of TB stigma among community members are shown in Table 3. After adjusting for other covariates, district showed the largest association with TB stigma (mean score difference: 5.73; 95% *CI* 2.19, 9.27), with community members from Zululand reporting stigma levels nearly six points higher than community members from BCM. Among those who ever had TB, stigma scores were more than two points lower compared to those who have never had TB (difference: − 2.19; 95% *CI* − 4.37, − 0.01). All other characteristics had only a one-point difference or less, except for community type (rural/farming vs small town; difference: − 2.81; 95% *CI* − 6.52, 0.91), but this did not reach statistical significance.

**Table 3:** Crude and multivariable analysis investigating correlates of community stigma scores among community members

Community members				
Variable	Crude analysis		Multivariable analysis	
	Community stigma score			
	$\beta$ (95% *CI*)	*P*-value	$\beta$ (95% *CI*)	*P*-value
District				
Zululand	3.43 (1.81, 5.06)	0.00	5.73 (2.19, 9.27)	*0.00*
Gender				
Male	0.68 (− 0.82, 2.17)	0.37	0.63 (− 0.82, 2.08)	0.39
Age	0.034 (− 0.026, 0.094)	0.27		
Race				
Black	− 0.51 (− 2.08, 1.05)	0.52		
Relationship				
Union	0.99 (− 0.54, 2.53)	0.20	1.09 (− 0.44, 2.62)	0.16
Employment				
Employed	0.20 (− 1.31, 1.70)	0.80		
Community type				
Rural/farming	2.55 (0.80, 4.30)	0.01	− 2.81 (− 6.52, 0.91)	0.14
Education				
12th grade and above	0.50 (− 1.01, 1.99)	0.52		
Income				
≥ ZAR 5000	0.28 (− 1.34, 1.89)	0.74		
Causes of TB*				
Good understanding	0.20 (− 1.44, 1.84)	0.37		
Mixed understanding	− 1.01 (− 3.24, 1.21)	0.81		
Poor understanding	REF			
TB/HIV knowledge				
TB increases chance of HIV	1.19 (− 0.30, 2.68)	0.12	0.56 (− 0.91, 2.04)	0.45
HIV increases chance of TB	− 1.78 (− 3.99, 0.44)	0.12	− 1.08 (− 3.31, 1.16)	0.34
TB contacts	− 0.087 (− 1.68, 1.50)	0.91		
Ever having TB	− 1.64 (− 1.59, 0.99)	0.15	− 2.19 (− 4.37, − 0.0064)	*0.05*
HIV status				
Positive	0.63 (− 1.71, 2.97)	0.56		
Negative	1.05 (− 0.93, 3.02)	0.30		
Unknown/no test	REF			
HIV stigma	− 0.028 (− 0.25, 0.19)	0.80		
Social support	− 0.060 (− 0.14, − 0.020)	0.14	− 0.035 (− 0.12, 0.048)	0.41

^*^Good understanding denotes participants who only identified accurate causes of TB (i.e. bacteria). Mixed understanding denotes participants who identified accurate and mis-informed causes of TB (i.e. infection and witchcraft). Poor understanding denotes participants who only identified incorrect causes of TB (i.e. exposure to the cold)

### Correlates of TB stigma among TB presumptives

Correlates of TB stigma among TB presumptives are shown in Table 4. After adjusting for other covariates, knowing that HIV increases the chances of TB was associated with higher TB stigma scores (score difference: 2.48; 95% *CI* 0.020, 4.94). Though not statistical significant, incorrectly believing that having TB increased the chances of HIV was also associated with higher TB stigma (score difference: 1.35; 95% *CI* − 0.36, 3.06). A higher social support score was associated with lower TB stigma (score difference: − 0.077; 95% *CI* − 0.14, − 0.01).

**Table 4:** Crude and multivariable analysis investigating correlates of community stigma scores among tuberculosis (TB) presumptives

TB presumptives				
Variable	Crude analysis		Multivariable analysis	
	Community stigma score			
	$\beta$ (95% *CI*)	*P*-value	$\beta$ (95% *CI*)	*P*-value
District				
Zululand	0.90 (− 0.75, 2.55)	0.28	1.02 (− 0.86, 2.89)	0.28
Gender				
Male	0.99 (− 0.55, 2.54)	0.21	0.0075 (− 1.54, 1.55)	0.99
Age	− 0.025 (− 0.092, 0.043)	0.47		
Race				
Black	− 0.57 (− 2.39, 1.25)	0.54		
Relationship				
Union	− 1.04 (− 2.73, 0.64)	0.22	− 0.81 (− 2.48, 0.85)	0.34
Employment				
Employed	0.37 (− 1.22, 1.96)	0.65		
Community type				
Rural/farming	0.89 (− 0.86, 2.63)	0.32		
Education				
12th grade and above	− 0.89 (− 2.45, 0.67)	0.26		
Income				
≥ ZAR 5000	− 0.26 (− 2.21, 1.69)	0.79		
Causes of TB*				
Good understanding	0.45 (− 1.23, 2.13)	0.60	− 0.23 (-2.00, 1.54)	0.80
Mixed understanding	− 1.59 (-4.01, 0.84)	0.20	− 1.28 (-3.60, 1.04)	0.28
Poor understanding	REF		REF	
TB/HIV knowledge				
TB increases chance of HIV	2.12 (0.62, 3.63)	0.01	1.35 (− 0.36, 3.06)	0.12
HIV increases chance of TB	2.90 (0.79, 5.02)	0.01	2.48 (0.020, 4.94)	*0.05*
TB contacts	− 1.37 (− 2.90, 0.17)	0.08	− 1.01 (− 2.58, 0.55)	0.20
Ever having TB	0.34 (− 1.68, 2.36)	0.74		
HIV status				
Positive	− 0.63 (− 3.00, 1.73)	0.60		
Negative	− 1.19 (− 3.49, 1.12)	0.31		
Unknown/no test	REF			
HIV stigma	0.036 (− 0.16, 0.23)	0.71		
Social support	− 0.093 (− 0.16, − 0.026)	0.01	− 0.077 (− 0.14, − 0.010)	*0.02*

^*^Good understanding denotes participants who only identified accurate causes of TB (i.e. bacteria). Mixed understanding denotes participants who identified accurate and mis-informed causes of TB (i.e. infection and witchcraft). Poor understanding denotes participants who only identified incorrect causes of TB (i.e. exposure to the cold)

Higher depression scores were associated with higher TB stigma (score difference: 0.15; 95% *CI* − 0.01, 0.31) when adjusting for covariates included in the primary multivariable model, though this was not statistical significance (see Additional file 2: Table S1); depression was not included in the primary analysis shown in Table 4 so as to be comparable with results from community participants for which depression was not assessed.

### Correlates of TB stigma among TB patients

Correlates of TB stigma among TB patients are shown in Table 5. Identifying as black (race) (score difference: − 2.90; 95% *CI* − 4.74, − 1.04) and having good (score difference: − 2.93; 95% *CI* − 4.92, − 0.94) or mixed (score difference: − 1.26; 95% CI − 2.82, 0.31) understanding of causes of TB as well as higher social support (score difference: − 0.054; 95% *CI* − 0.12, 0.016) were associated with lower TB stigma. Conversely, believing that TB increases chances of HIV (score difference: 2.48; 95% *CI* 1.05, 3.90) and higher HIV stigma (score difference: 0.32; 95% *CI* 0.21, 0.42) were associated with higher TB stigma. When including depression in the multivariable model, higher scores were associated with higher TB stigma (score difference: 0.21; 95% *CI* 0.030, 0.38) (see Additional file 2: Table S2).

**Table 5:** Crude and multivariable analysis investigating correlates of community stigma scores among tuberculosis (TB) patients

TB patients				
Variable	Crude analysis		Multivariable analysis	
	Community stigma score			
	$\beta$ (95% *CI*)	*P*-value	$\beta$ (95% *CI*)	*P*-value
District				
Zululand	2.47 (0.74, 4.75)	0.01	0.99 (− 2.71, 4.69)	0.60
Gender				
Male	− 0.52 (− 2.51, 1.47)	0.61	− 0.64 (− 1.99, 0.71)	0.46
Age	0.013 (-0.077, 0.10)	0.78		
Race				
Black	− 2.44 (− 4.71, − 0.18)	0.03	− 2.90 (− 4.74, − 1.04)	*0.00*
Relationship				
Union	0.72 (− 1.38, 2.82)	0.50		
Employment				
Employed	− 0.22 (− 2.23, 1.79)	0.83		
Community type				
Rural/farming	3.81 (1.81, 5.81)	0.00	1.30 (− 2.70, 5.30)	0.52
Education				
12th grade and above	− 0.14 (− 2.22, 1.93)	0.89		
Income				
≥ ZAR 5000	− 2.90 (− 5.33, − 0.48)	0.02	0.20 (− 1.56, 1.95)	0.83
Causes of TB*				
Good understanding	− 1.64 (− 3.65, 0.37)	0.11	− 1.26, (− 2.82, 0.31)	0.11
Mixed understanding	− 6.33 (− 8.46, -4.02)	0.00	− 2.93 (− 4.92, − 0.94)	*0.00*
Poor understanding	REF		REF	
TB/HIV knowledge				
TB increases chance of HIV	4.42 (2.61, 6.24)	0.00	2.48 (1.05, 3.90)	*0.00*
HIV increases chance of TB	0.47 (− 2.30, 3.25)	0.74		
TB contacts	169 (− 0.33, 3.72)	0.10	0.70 (− 0.69, 2.10)	0.32
Ever having TB	0.47 (− 2.08, 3.00)	0.72		
HIV status				
Positive	− 1.12 (− 4.27, 2.04)	0.49		
Negative	0.36 (− 2.89, 3.61)	0.83		
Unknown/no test	REF			
HIV stigma	0.38 (0.26, 0.50)	0.00	0.32 (0.21, 0.42)	*0.00*
Social support	− 0.22 (− 0.30, − 0.14)	0.00	− 0.054 (− 0.12, 0.016)	0.13

^*^Good understanding denotes participants who only identified accurate causes of TB (i.e. bacteria). Mixed understanding denotes participants who identified accurate and mis-informed causes of TB (i.e. infection and witchcraft). Poor understanding denotes participants who only identified incorrect causes of TB (i.e. exposure to the cold)

### Summary of correlated across cadres

The results from all three models across participant types are summarized in Table 6. No single characteristic was found to be associated with TB stigma across all participant types. TB knowledge generally associated with TB stigma for both presumptives and patients, as did social support and depression. TB patients had the largest number of different characteristics found to be associated with TB stigma.

**Table 6:** Summary table of associations within multivariate analyses for community members, tuberculosis presumptives, and tuberculosis patients

	Community members	TB presumptives	TB patients
District: Zululand	++		
Gender: Male			
Age	XXX	XXX	XXX
Race: Black	XXX	XXX	−−
Relationship: Union			XXX
Community type: Rural/farming	–	XXX	
Income: ≥ ZAR 5000	XXX	XXX	
Cause of TB: Good understanding	XXX		–
Cause of TB: Mixed understanding	XXX		−−
TB increases chance of HIV		+	++
HIV increases chance of TB		++	XXX
TB contact	XXX		
Ever have TB	−−	XXX	XXX
HIV stigma	XXX	XXX	++
Social support		−−	–
Depression	n/a	+	++

All results are from models that exclude depression, except the depression result (adjusted for all others listed)

^+^*P* < 0.15;^++^*P* < 0.05 (same for negative associations; depression in presumptives was only result that was *P* < 0.10); white box indicates *P* ≥ 0.15; XXX indicates not selected for multivariable (crude *P* > 0.25); n/a = not asked of community members

## Discussion

This analysis sought to quantify, using validated TB stigma measures, the presence, level, and correlates of TB stigma amongst three important groups: community members, TB presumptives, and TB patients. We found that TB presumptives had the highest levels of TB stigma compared to both community members and TB patients. Furthermore, distinct correlates were associated with TB stigma at different points along the TB cascade, implying that stigma is dynamic and influenced by different factors along the TB cascade. These findings provide important insights for future studies aimed at understanding the types and levels of stigma present along the TB cascade, how stigma may differentially impact different cadres of individuals engaged along the TB cascade, the impact of stigma on TB outcomes, and how TB stigma may intersect with other stigmatizing characteristics (i.e., gender, HIV status, poverty).

TB presumptives sit at the crucial entry point of the TB treatment cascade. Their disengagement from care before receiving their test results and initiating TB treatment if TB positive, may lead to further TB transmission at home and in the community. Yet few studies have focused on quantitatively investigating the presence and effect of TB stigma among TB presumptives. There is a varied and contradictory body of knowledge surrounding TB stigma among TB presumptives [26, 41]. A study among TB presumptives from rural Southwest Ethiopia found no associations with TB stigma among TB presumptives [41]. Another study found that gender and low levels of general TB knowledge (i.e. transmission, symptoms, treatment, and comorbidities) were associated with increased TB stigma [26]. These findings could result from differences in population context, study design, and the use of invalidated stigma measures. To overcome these inconsistencies, a large-scale longitudinal study must be conducted utilizing validated stigma scales which measure specific types of TB stigma (i.e. disclosure, anticipated, isolation). The longitudinal design will allow researchers to determine how stigma impacts outcomes, while use of valid scales will produce a more accurate measure. Future research should explore, in a prospective manner, the extent to which stigma impacts initial loss to follow up among presumptives. If found to be associated, targeted stigma reduction interventions should be developed to improve retention along the testing cascade and treatment initiation.

Among TB presumptives, we also found that having correct knowledge of the role of HIV in TB disease was associated with a sizable increase in TB stigma. Interestingly, Godfrey-Faussett et al. reported that in Zambia low levels of general TB knowledge (i.e. transmission, symptoms, treatment, and comorbidities) was associated with increased TB stigma [26]. While these discrepancies may be due in part to the contextual setting of the study population and the ambiguity of the umbrella term “TB Knowledge”, qualitative research has highlighted community perceptions that a TB diagnosis means that an individual also has HIV [22, 39]. Knowing that a TB diagnosis is perceived as a marker for HIV positivity, TB presumptives may anticipate detrimental effects on their social status and relationships. This, in turn, may result in their delaying or forgoing presentation for TB test results. Ultimately, the social intricacies of being diagnosed with TB in high HIV-burden settings may influence one’s health behavior during this intersection of the cascade. Further investigating the association between TB and HIV diagnoses and its impact on TB presumptives should be conducted to determine how perceived stigma may impact TB presumptives initial loss to follow up.

More often, studies of TB stigma have focused on correlates among community members or patients. Among community members in Ethiopia, education, TB knowledge, and region of country were found to be associated with TB stigma [50]. Although these results may reflect contextual differences, we could not discern an association between level of education or TB knowledge and TB stigma among community members in our study. However, we did find a substantial association between health districts and stigma (5.73 points higher in Zululand compared to BCM). Furthermore, previously experiencing TB reduced TB stigma levels (2.19 points lower). While these results may suggest that health stigma is context dependent, and its levels and presence varies depending on an individual’s experiences [7, 8, 10, 51], these results could also highlight that differences may be due to differences in measurement tools. In fact, a study conducted in Thailand using the same Van Rie TB stigma measure as this study, similarly found that gender, income, TB contacts, and knowledge of HIV being associated with TB were not associated with stigma [52]. Unlike our study, the Thailand study did find both older age and HIV stigma to be associated with TB stigma among community members.

In Zambia and Sudan, observational studies of TB patients reported gender, age, education, geographical location, employment, and TB awareness to be associated with TB stigma [39, 53]. Among TB patients in our study, knowing the correct causes of TB, knowledge of the role of TB in HIV, perceived HIV stigma and race/ethnic group were associated with TB stigma. Specifically, understanding the correct causes of TB decreased stigma scores among TB patients by 2.93 points. Research has shown that among TB patients, an increase in general understanding of TB may disprove incorrect rumors and information about TB that would have otherwise increased stigma [15, 54]. Among TB patients reporting higher levels of HIV stigma and knowing that TB increases the chances of HIV, there was also a significant association with reporting higher levels of TB stigma. This may highlight the issue of intersectional stigma – creating an additional layer of stigma that is the transfer of stigmatizing beliefs previously linked to HIV and TB [55]. Interestingly, this results was also reported among TB patients in Thailand who were administered the same validated Van Rie perceived TB stigma scale [52]. Finally, regarding race, TB patients self-identifying as black reported lower stigma scores (2.90 points lower) compared to those self-identifying as non-black. In our study population, 77.0% of non-black TB patients identify as Coloured. South Africa categorizes as “Coloured” the multiracial ethnic group native to Southern Africa who have ancestry from more than one of the various populations inhabiting the region, including Khoisan, Bantu, Afrikaner, Whites, Austronesian, East Asian or South Asian [56, 57]. In this context, while race is likely a proxy for cultural/ethnic group identity, it may also be a proxy for geographic location. Specifically, black African participants were recruited from Zululand district while all Coloured participants were recruited from BCM district. Given these finding, understanding the intersectionality of TB and HIV stigma, as well as South African racial/ethnic groupings and TB stigma deserved further exploration.

The limitations of this study should be noted. First, the Van Rie Perceived Community TB stigma scale had not been previously used among TB presumptives, nor in the South African context. The scale was intended to measure the perceptions of how community members feel about people who have TB and had previously been used among both community members and TB patients in Thailand [52]. Nevertheless, we found it had good performance in the South Africa context including among TB presumptives [49], but more research is needed on TB measures in TB presumptives. A second limitation was the small, cross-sectional nature of the study, which may have suffered from low power to identify some factors associated with TB stigma. We attempted to minimize this by selecting model variables with a *P*-value ≤ 0.25 and by reporting associations where *P* < 0.15 in order to reduce type II errors. Furthermore, because stigma is time and context dependent and needs to be analyzed over a period of time, we were unable to properly measure assumption of causality between the correlates and stigma levels. A prospective, longitudinal study involving participants progressing along the TB cascade, from presentation for testing through to treatment completion, would provide key and actionable insight into how TB stigma changes as individuals progress along the TB cascade, and stigma’s associations with and effects on TB outcomes. Additionally, future analyses that seek to further investigate the causal effect of specific factors identified in this paper should use an appropriate causal framework within the TB cascade and appropriate statistical methods for causal inference. A third limitation was participant selection. Specifically, all participants were recruited from public healthcare facilities. To the extent that individuals with higher TB stigma avoid healthcare facilities, our results may suffer from selection bias, skewing our data to show overall lower levels of stigma than what may actually exist within these larger communities.

The main strength of this study was its ability to capture data from a diverse population within South Africa while utilizing a validated TB stigma measure. The use of a validated tool to measure and quantify an abstract concept such as stigma increase the validity and reliability of the results while upholding high quality research. This study also examined TB stigma among TB patients, TB presumptives, and community members, allowing for a comparison beyond the classic silo of TB patients or community member which is seen in the majority of quantitative TB stigma studies. Understanding the differences between these three cadres may allow for distinct approaches to reduce TB stigma at different points along the TB cascade.

## Conclusions

Tuberculosis remains a pressing health concern in South Africa. Stigma is likely an important, but underexplored and underappreciated factor impacting global progress towards ending the global TB epidemic. This study further highlights the importance of considering TB presumptives when designing stigma interventions and understanding the intricacies of movement along the TB cascade. Furthermore, it highlights the fact that stigma is driven by a variety of factors experienced at different points along the TB cascade leading to the need for tailored stigma reduction programs. Understanding stigma and how it interacts with patient progression along the TB cascade will provide further insight into specific subpopulations, especially those at greater risk for poor TB outcomes.

## Supplementary information

**Additional file 1.** Questionnaire.

**Additional file 2: Table S1.** Crude and multivariable analysis investigating correlates of community stigma scores among tuberculosis (TB) presumptives including mental health. **Table S2.** Crude and multivariable analysis investigating correlates of community stigma scores among tuberculosis (TB) patients including mental health.

## Data Availability

The datasets used and analysed during the current study are available from the corresponding author on reasonable request.

## Abbreviations

TB: Tuberculosis
HIV: Human immunodeficiency virus
SA-NTP: South African National TB Programme
BCM: Buffalo City Metro
