# Supplementary material for: Quantification and correlates of tuberculosis stigma along the tuberculosis testing and treatment cascades in South Africa: a cross-sectional study
Source: Infect Dis Poverty. 2020 Oct 22;9:145. doi: 10.1186/s40249-020-00762-8 (PMC7579945; doi:10.1186/s40249-020-00762-8)
Supplement: Supplementary file 1 — Additional file 1. Questionnaire. [file 40249_2020_762_MOESM1_ESM.pdf]

TB STIGMA CODE BOOK

#	Field Label	Field Attributes
**Sociodemographics**		
1	District	1= BCM<br>2= Zululand
2	What is your race?	1= White<br>2= Black<br>3= Indian<br>4= Colored<br>5= Other, specify
3	Are you male or female?	1= Female<br>2=Male
4	What is your relationship status?	1= Single<br>2= In a relationship, but unmarried (living together)<br>3= Married<br>4= Separated<br>5= Divorced<br>6= Widowed
5	What is the highest level of education that you have completed?	1= None<br>2= Grade 7 (primary)<br>3= Grade 8 to 11 (before matric)<br>4= Grade 12<br>5= Tertiary
6	What is your employment status?	1= Employed full-time<br>2= Employed part-time<br>3= Self-employed<br>4= Unemployed
7	Where do you stay?	1= Rural<br>2= Farming area<br>3= Small town<br>4= Large city
8	Who do you currently stay with?	1= Family<br>2= Living with friends<br>3= Living alone
9	Where do you get money to meet your basic monthly needs?	1= Government grant<br>2= Salary/Wages from employer<br>3= Profits from own business<br>4= Partner/spouse<br>5= Children<br>6= Family
10	What is your total household income per month?	1= Under R5000<br>2= R5000 – R10000<br>3= R10000 – R20000<br>4= R20000 – R30000<br>5= More than R30000<br>6= Don't want to answer
11	Have you ever had TB in the past?	1= Never<br>2= Yes, less than 2 years ago<br>3= Yes, more than 2 years ago
12	Do you know anyone who currently has TB?	0= No<br>1= Yes
13	Have you been tested for HIV?	0= No<br>1= Yes<br>3= I don't know
14	Would you mind telling us the results?	1= Positive<br>2= Negative<br>3= Does not want to disclose
**Multidimensional Scale of Social Support**		

15	There is a special person who is around when I am in need.	1= Very strongly disagree<br>2= Strongly disagree<br>3= Mildly disagree<br>4= Neutral<br>5= Mildly agree<br>6= Strongly agree<br>7= Very strongly agree
16	There is a special person whom I can share joys and sorrows with	1= Very strongly disagree<br>2= Strongly disagree<br>3= Mildly disagree<br>4= Neutral<br>5= Mildly agree<br>6= Strongly agree<br>7= Very strongly agree
17	My family really tries to help me.	1= Very strongly disagree<br>2= Strongly disagree<br>3= Mildly disagree<br>4= Neutral<br>5= Mildly agree<br>6= Strongly agree<br>7= Very strongly agree
18	I get the emotional help & support I need from my family.	1= Very strongly disagree<br>2= Strongly disagree<br>3= Mildly disagree<br>4= Neutral<br>5= Mildly agree<br>6= Strongly agree<br>7= Very strongly agree
19	I have a special person who is a real source of comfort to me.	1= Very strongly disagree<br>2= Strongly disagree<br>3= Mildly disagree<br>4= Neutral<br>5= Mildly agree<br>6= Strongly agree<br>7= Very strongly agree
20	My friends really try to help me.	1= Very strongly disagree<br>2= Strongly disagree<br>3= Mildly disagree<br>4= Neutral<br>5= Mildly agree<br>6= Strongly agree<br>7= Very strongly agree
21	I can count on my friends when things go wrong.	1= Very strongly disagree<br>2= Strongly disagree<br>3= Mildly disagree<br>4= Neutral<br>5= Mildly agree<br>6= Strongly agree<br>7= Very strongly agree
22	I can talk about my problems with my family.	1= Very strongly disagree<br>2= Strongly disagree<br>3= Mildly disagree<br>4= Neutral<br>5= Mildly agree<br>6= Strongly agree<br>7= Very strongly agree
23	I have friends with whom I can share my joys and sorrows.	1= Very strongly disagree<br>2= Strongly disagree<br>3= Mildly disagree<br>4= Neutral

		5= Mildly agree<br>6= Strongly agree<br>7= Very strongly agree
24	There is a special person in my life who cares about my feelings.	1= Very strongly disagree<br>2= Strongly disagree<br>3= Mildly disagree<br>4= Neutral<br>5= Mildly agree<br>6= Strongly agree<br>7= Very strongly agree
25	My family is willing to help me make decisions.	1= Very strongly disagree<br>2= Strongly disagree<br>3= Mildly disagree<br>4= Neutral<br>5= Mildly agree<br>6= Strongly agree<br>7= Very strongly agree
26	I can talk about my problems with my friends.	1= Very strongly disagree<br>2= Strongly disagree<br>3= Mildly disagree<br>4= Neutral<br>5= Mildly agree<br>6= Strongly agree<br>7= Very strongly agree
**HIV Stigma Scale (Kalichman 2005)**		
27	People who have AIDS are dirty	0= Strongly disagree<br>1= Disagree<br>2= Agree<br>3= Strongly agree
28	People who have AIDS are cursed	0= Strongly disagree<br>1= Disagree<br>2= Agree<br>3= Strongly agree
29	People who have AIDS should be ashamed	0= Strongly disagree<br>1= Disagree<br>2= Agree<br>3= Strongly agree
30	It is safe for people with AIDS to work with children	0= Strongly disagree<br>1= Disagree<br>2= Agree<br>3= Strongly agree
31	People with AIDS must expect some restrictions on their freedom	0= Strongly disagree<br>1= Disagree<br>2= Agree<br>3= Strongly agree
32	A person with AIDS must have done something wrong and deserves to be punished	0= Strongly disagree<br>1= Disagree<br>2= Agree<br>3= Strongly agree
33	People with HIV must be isolated	0= Strongly disagree<br>1= Disagree<br>2= Agree<br>3= Strongly agree
34	I do not want to be friends with someone who has AIDS	0= Strongly disagree<br>1= Disagree<br>2= Agree<br>3= Strongly agree
35	People with AIDS should not be allowed to work	0= Strongly disagree<br>1= Disagree<br>2= Agree

		3= Strongly agree
**Attributable HIV Stigma (Visser 2008)**		
36	Most people think that getting HIV is a punishment for bad behaviour	0= Strongly disagree<br>1= Disagree<br>2= Agree<br>3= Strongly agree
37	Most people think that having HIV is just a matter of bad luck	0= Strongly disagree<br>1= Disagree<br>2= Agree<br>3= Strongly agree
38	Most people think less of someone because they have HIV	0= Strongly disagree<br>1= Disagree<br>2= Agree<br>3= Strongly agree
39	Most people would not like someone with HIV to be living next door	0= Strongly disagree<br>1= Disagree<br>2= Agree<br>3= Strongly agree
40	Most people would reject the friendship of someone with HIV	0= Strongly disagree<br>1= Disagree<br>2= Agree<br>3= Strongly agree
41	Most people feel that it is safe for a person with HIV to look after somebody else's children	0= Strongly disagree<br>1= Disagree<br>2= Agree<br>3= Strongly agree
42	Most people would not date a person if they know that he/she has HIV	0= Strongly disagree<br>1= Disagree<br>2= Agree<br>3= Strongly agree
43	Most people are afraid to be around people with HIV	0= Strongly disagree<br>1= Disagree<br>2= Agree<br>3= Strongly agree
44	Most people feel that if you have HIV it is your own fault	0= Strongly disagree<br>1= Disagree<br>2= Agree<br>3= Strongly agree
45	Most employers would not hire someone with HIV to work for them	0= Strongly disagree<br>1= Disagree<br>2= Agree<br>3= Strongly agree
46	Most people would not drink from a tap if a person with HIV had just drunk from it	0= Strongly disagree<br>1= Disagree<br>2= Agree<br>3= Strongly agree
47	Most people believe that if you have HIV you must have done something wrong to deserve it	0= Strongly disagree<br>1= Disagree<br>2= Agree<br>3= Strongly agree
48	Most people believe that someone with HIV should be ashamed of themselves	0= Strongly disagree<br>1= Disagree<br>2= Agree<br>3= Strongly agree
49	Most people feel uncomfortable around people with HIV	0= Strongly disagree<br>1= Disagree<br>2= Agree<br>3= Strongly agree
**Mental Health Assessment (PHQ8)**		
50	Little interest or pleasure in doing things	0= Not at all

		1= Several days<br>2= More than half the days<br>3= Nearly every day
51	Feeling down, depressed or hopeless	0= Not at all<br>1= Several days<br>2= More than half the days<br>3= Nearly every day
52	Trouble falling asleep, staying asleep, or sleeping too much	0= Not at all<br>1= Several days<br>2= More than half the days<br>3= Nearly every day
53	Feeling tired or having little energy	0= Not at all<br>1= Several days<br>2= More than half the days<br>3= Nearly every day
54	Poor appetite or overeating	0= Not at all<br>1= Several days<br>2= More than half the days<br>3= Nearly every day
55	Feeling bad about yourself - or that you're a failure or have let yourself or your family down	0= Not at all<br>1= Several days<br>2= More than half the days<br>3= Nearly every day
56	Trouble concentrating on things, such as reading the newspaper or watching television	0= Not at all<br>1= Several days<br>2= More than half the days<br>3= Nearly every day
57	Moving or speaking so slowly that other people could have noticed. Or, the opposite - being so fidgety or restless that you have been moving around a lot more than usual	0= Not at all<br>1= Several days<br>2= More than half the days<br>3= Nearly every day
**TB Knowledge**		
58	What do you think causes TB?	1= I don’t know<br>2= Infection/Virus/Bacteria<br>3= Witchcraft<br>4= Infection from others<br>5= Smoking<br>6= Exposure to the cold<br>7= Drinking alcohol<br>8= Weak body<br>9= Other, specify
59	How is TB spread from person to person?	1= I don’t know<br>2= Physical contact (shaking hands, kissing, and so on)<br>3= Sharing foods/drinks/utensils<br>4= Coughing/sneezing/talking/singing<br>5= Poor sanitation/sharing toilet seats<br>6= Sex<br>7= Other, specify
60	What are the signs and symptoms of TB?	1= I don’t know<br>2= Fever<br>3= Weight loss<br>4= Coughing<br>5= Night sweats<br>6= Coughing up blood<br>7= Loss of appetite<br>8= Chest pain<br>9= Breathlessness<br>10 = Feeling weak<br>11= Other, specify

61	Can TB be cured?	0= No<br>1= Yes<br>3= I don't know
62	Does having HIV/AIDS increase the chances of getting TB?	0= No<br>1= Yes<br>3= I don't know
63	Does having TB increase the chances of having HIV/AIDS?	0= No<br>1= Yes<br>3= I don't know
	**Van Rie Community TB Stigma**	
64	People may not want to eat or drink with friends who have TB	0= Strongly disagree<br>1= Disagree<br>2= Agree<br>3= Strongly agree
65	People may not want to eat or drink with relatives who have TB	0= Strongly disagree<br>1= Disagree<br>2= Agree<br>3= Strongly agree
66	People feel uncomfortable about being near those with TB	0= Strongly disagree<br>1= Disagree<br>2= Agree<br>3= Strongly agree
67	People do not want those with TB playing with their children	0= Strongly disagree<br>1= Disagree<br>2= Agree<br>3= Strongly agree
68	People keep their distance from people with TB	0= Strongly disagree<br>1= Disagree<br>2= Agree<br>3= Strongly agree
69	People do not want to talk to others with TB	0= Strongly disagree<br>1= Disagree<br>2= Agree<br>3= Strongly agree
70	People are afraid of those with TB	0= Strongly disagree<br>1= Disagree<br>2= Agree<br>3= Strongly agree
71	People try not to touch others with TB	0= Strongly disagree<br>1= Disagree<br>2= Agree<br>3= Strongly agree
