# Supplementary material for: Quantification and correlates of tuberculosis stigma along the tuberculosis testing and treatment cascades in South Africa: a cross-sectional study
Source: Infect Dis Poverty. 2020 Oct 22;9:145. doi: 10.1186/s40249-020-00762-8 (PMC7579945; doi:10.1186/s40249-020-00762-8)
Supplement: Supplementary file 2 — Additional file 2: Table S1. Crude and multivariable analysis investigating correlates of community stigma scores among tuberculosis (TB) presumptives including mental health. Table S2. Crude and multivariable analysis investigating correlates of community stigma scores among tuberculosis (TB) patients including mental health. [file 40249_2020_762_MOESM2_ESM.docx]

**Additional file:**

**S1:** Crude and multivariable analysis investigating correlates of community stigma scores among tuberculosis (TB) presumptives including mental health

| TB presumptives | | | | | |
| --- | --- | --- | --- | --- | --- |
| Variable | | Crude analysis | | Multivariable analysis | |
|  |  | **Community stigma score** | | | |
|  |  | $\beta$ (95% *CI*) | *P-value* | $\beta$ (95% *CI*) | *P-value* |
| District | Zululand | 0.90 (-0.75, 2.55) | 0.28 | 0.55 (-1.37, 2.47) | 0.57 |
| Gender | Male | 0.99 (-0.55, 2.54) | 0.21 | -0.017 (-1.54, 1.51) | 0.98 |
| Age | | -0.025 (-0.092, 0.043) | 0.47 |  |  |
| Race | Black | -0.57 (-2.39, 1.25) | 0.54 |  |  |
| Relationship | Union | -1.04 (-2.73, 0.64) | 0.22 | -0.86 (-2.51, 0.79) | 0.30 |
| Employment | Employed | 0.37 (-1.22, 1.96) | 0.65 |  |  |
| Community type | Rural/farming | 0.89 (-0.86, 2.63) | 0.32 |  |  |
| Education | 12^th^ grade & above | -0.89 (-2.45, 0.67) | 0.26 |  |  |
| Income | ≥ ZAR 5000 | -0.26 (-2.21, 1.69) | 0.79 |  |  |
| Causes of TB | Poor understanding | 0.45 (-1.23, 2.13) | 0.60 | -0.41 (-2.17, 1.35) | 0.65 |
|  | Mixed understanding | -1.59 (-4.01, 0.84) | 0.20 | -1.60 (-3.92, 0.72) | 0.18 |
|  | Good understanding | REF | |  |  |
| TB/HIV Knowledge | TB increases chance of HIV | 2.12 (0.62, 3.63) | 0.01 | 1.18 (-0.52, 2.88) | 0.17 |
|  | HIV increases chance of TB | 2.90 (0.79, 5.02) | 0.01 | 2.62 (0.18, 5.06) | 0.04 |
| TB contacts | | -1.37 (-2.90, 0.17) | 0.08 | -0.75 (-2.33, 0.83) | 0.35 |
| Ever having TB | | 0.34 (-1.68, 2.36) | 0.74 |  |  |
| HIV status | Positive | -0.63 (-3.00, 1.73) | 0.60 |  |  |
|  | Negative | -1.19 (-3.49, 1.12) | 0.31 |  |  |
|  | Unknown/No test | REF | | REF | |
| HIV stigma | | 0.036 (-0.16, 0.23) | 0.71 |  |  |
| Mental health | | 0.18 (0.034, 0.33) | 0.02 | 0.15 (-0.0096, 0.31) | 0.07 |
| Social support | | -0.093 (-0.16, -0.026) | 0.01 | -0.071 (-0.14, -0.0049) | 0.04 |

**S2:** Crude and multivariable analysis investigating correlates of community stigma scores among tuberculosis (TB) patients including mental health

| TB patients | | | | | |
| --- | --- | --- | --- | --- | --- |
| Variable | | Crude analysis | | Multivariable analysis | |
|  |  | Community stigma score | | | |
|  |  | $\beta$ (95% *CI*) | *P-value* | $\beta$ (95% *CI*) | *P-value* |
| District | Zululand | 2.47 (0.74, 4.75) | 0.01 | 0.75 (-2.88, 4.37) | 0.68 |
| Gender | Male | -0.52 (-2.51, 1.47) | 0.61 | -0.40 (-1.74, 0.94) | 0.55 |
| Age | | 0.013 (-0.077, 0.10) | 0.78 |  |  |
| Race | Black | -2.44 (-4.71, -0.18) | 0.03 | -2.82 (-4.64, -1.01) | 0.00 |
| Relationship | Union | 0.72 (-1.38, 2.82) | 0.50 |  |  |
| Employment | Employed | -0.22 (-2.23, 1.79) | 0.83 |  |  |
| Community type | Rural/farming | 3.81 (1.81, 5.81) | 0.00 | 0.63 (-3.32, 4.57) | 0.75 |
| Education | 12^th^ grade & above | -0.14 (-2.22, 1.93) | 0.89 |  |  |
| Income | ≥ ZAR 5000 | -2.90 (-5.33, -0.48) | 0.02 | 0.12 (-1.59, 1.84) | 0.89 |
| Causes of TB | Poor understanding | -1.64 (-3.65, 0.37) | 0.11 | -1.22, (-2.75, 0.31) | 0.12 |
|  | Mixed understanding | -6.33 (-8.46, -4.02) | 0.00 | -2.64 (-4.60, -0.69) | 0.01 |
|  | Good understanding | REF | | REF | |
| TB/HIV Knowledge | TB increases chance of HIV | 4.42 (2.61, 6.24) | 0.00 | 2.30 (0.90, 3.70) | 0.00 |
|  | HIV increases chance of TB | 0.47 (-2.30, 3.25) | 0.74 |  |  |
| TB contacts | | 169 (-0.33, 3.72) | 0.10 | 0.65 (-0.71, 2.01) | 0.35 |
| Ever having TB | | 0.47 (-2.08, 3.00) | 0.72 |  |  |
| HIV status | Positive | -1.12 (-4.27, 2.04) | 0.49 |  |  |
|  | Negative | 0.36 (-2.89, 3.61) | 0.83 |  |  |
|  | Unknown/No test | REF | |  | |
| HIV stigma | | 0.38 (0.26, 0.50) | 0.00 | 0.30 (0.20, 0.40) | 0.00 |
| Mental health | | 0.55 (0.35, 0.74) | 0.00 | 0.21 (0.030, 0.38) | 0.02 |
| Social support | | -0.22 (-0.30, -0.14) | 0.00 | -0.048 (-0.12, 0.021) | 0.17 |
